# Cell-Type-Specific Afferent Innervation of the Nucleus Accumbens Core and Shell

**DOI:** 10.3389/fnana.2018.00084

**Published:** 2018-10-16

**Authors:** Zhao Li, Zhilong Chen, Guoqing Fan, Anan Li, Jing Yuan, Tonghui Xu

**Affiliations:** ^1^Britton Chance Center for Biomedical Photonics, Wuhan National Laboratory for Optoelectronics, Huazhong University of Science and Technology, Wuhan, China; ^2^MoE Key Laboratory for Biomedical Photonics, Collaborative Innovation Center for Biomedical Engineering, School of Engineering Sciences, Huazhong University of Science and Technology, Wuhan, China

**Keywords:** nucleus accumbens core and shell, cell-type-specific, rabies virus, whole-brain inputs, quantitative analyses

## Abstract

The nucleus accumbens (NAc) is clearly implicated in reward processing and drug addiction, as well as in numerous neurological and psychiatric disorders; nevertheless, the circuit mechanisms underlying the diverse functions of the NAc remain poorly understood. Here, we characterized the whole-brain and monosynaptic inputs to two main projection cell types – D1 dopamine receptor expressing medium spiny neurons (D1R-MSNs) and D2 dopamine receptor expressing medium spiny neurons (D2R-MSNs) – within the NAc core and NAc shell by rabies-mediated trans-synaptic tracing. We discovered that D1R-MSNs and D2R-MSNs in both NAc subregions receive similar inputs from diverse sources. Inputs to the NAc core are broadly scattered, whereas inputs to the NAc shell are relatively concentrated. Furthermore, we identified numerous brain areas providing important contrasting inputs to different NAc subregions. The anterior cortex preferentially innervates the NAc core for both D1R-MSNs and D2R-MSNs, whereas the lateral hypothalamic area (LH) preferentially targets D1R-MSNs in the NAc shell. Characterizing the cell-type-specific connectivity of different NAc subregions lays a foundation for studying how diverse functions of the NAc are mediated by specific pathways.

## Introduction

The nucleus accumbens (NAc), a brain region located in the ventral aspect of the basal ganglia, is widely recognized to act as a limbic-motor interface (Floresco, 2015). The NAc has been implicated in various important functions including reward-related processes (Ambroggi et al., 2008; Carlezon and Thomas, 2009; Day et al., 2011; Russo and Nestler, 2013), spatial navigation (Floresco et al., 1997; Ito et al., 2008), feeding (Krause et al., 2010; Kenny, 2011; Urstadt et al., 2013), and sexual motivation (Everitt, 1990; Beny-Shefer et al., 2017). In addition, dysfunction of this region is thought to be involved in numerous neurological and psychiatric disorders such as depression (Russo and Nestler, 2013; Francis and Lobo, 2017), anxiety disorders (Fu et al., 2017), Alzheimer’s disease (Ferrier et al., 1983; Nie et al., 2017), Parkinson’s disease (Albin et al., 1989; DeLong, 1990), and drug addiction (Russo et al., 2010; Lüscher, 2016). Previous studies have established that the NAc integrates inputs from many brain areas, such as the ventral midbrain (Fallon and Moore, 1978; Phillipson and Griffiths, 1985), basal amygdaloid complex (Wright et al., 1996; Groenewegen et al., 1999), hippocampus (Kelley and Domesick, 1982; Lopes da Silva et al., 1984; Yang and Mogenson, 1984), thalamus (Phillipson and Griffiths, 1985; Berendse and Groenewegen, 1990; Brog et al., 1993), and prelimbic and prefrontal cortex (Beckstead, 1979; Phillipson and Griffiths, 1985; Montaron et al., 1996; Gorelova and Yang, 1997). However, how the diverse functions of the NAc are regulated by these complex anatomical connections remains poorly understood.

The NAc, however, is an inhomogeneous tissue that is often divided into two primary segments: the shell and the core (Zahm and Brog, 1992). The functions of these two subregions differ significantly in many behavioral paradigms. For example, approach toward reward-related stimuli is mediated by the NAc core but not the shell (Parkinson et al., 2000; Di Ciano et al., 2001; Salamone and Correa, 2012), whereas the opposite holds true for learning about the irrelevance of stimuli (Gal et al., 2005; Floresco et al., 2006). Additionally, the projection neurons within the NAc are GABAergic medium spiny neurons (MSNs), which are dichotomized based on their expression of either D1 or D2 dopamine receptors (Gerfen et al., 1990; Kawaguchi, 1997). These two MSN subtypes, which together make up >95% of all NAc neurons, exert balanced but antagonistic roles in reinforcement learning. In visual cue-based reward learning, NAc (core + shell) D1R-MSNs are critical for learning acquisition, whereas D2R-MSNs are needed for the flexibility to the learning switch (Yawata et al., 2012). Opposing roles for these two MSN subtypes in learning is further supported by the finding that optogenetic activation of D2R-MSNs suppresses cocaine reward, with opposite effects induced by activation of D1R-MSNs (Lobo et al., 2010).

Overall, to dissect the diverse functions of NAc, it is crucial to determine the direct synaptic inputs to different anatomical subregions in a cell-type-specific manner. Previous studies using traditional tracers have provided valuable information about the connections of the NAc (Kelley and Domesick, 1982; Berendse and Groenewegen, 1990; Brog et al., 1993; Wright et al., 1996; Gorelova and Yang, 1997). However, technical limitations of these traditional methods make it difficult to map the whole-brain inputs to specific neuron types. Furthermore, traditional tracers may be taken up by fibers that pass by the NAc, resulting in nonspecific labeling. Recently, the development of monosynaptic retrograde transsynaptic tracing based on modified rabies virus (RV) has greatly facilitated the mapping of whole-brain inputs to genetically defined cell types (Wickersham et al., 2007; Watabe-Uchida et al., 2012; Pollak Dorocic et al., 2014; Weissbourd et al., 2014; Do et al., 2016).

In the present study, we utilized such a virus-based labeling technique to identify monosynaptic inputs to D1R- and D2R-MSNs within different NAc subregions. We then compared the input distribution patterns among four groups defined by cell type and location (i.e., D1R-MSNs in the NAc core, D1R-MSNs in the NAc shell, D2R-MSNs in the NAc core and D2R-MSNs in the NAc shell). Although the input distributions of different cell types were highly similar, different NAc subregions had significantly different biased inputs from numerous brain areas. These findings provide an anatomical foundation for future studies of the neural circuits that underlie the diverse functions of the NAc.

## Materials and Methods

### Animals

All the experimental procedures were approved by the Hubei Provincial Animal Care and Use Committee and the experimental guidelines of the Animal Experimentation Ethics Committee of Huazhong University of Science and Technology. BAC-transgenic D1R-Cre [MMRRC Tg (Drd1acre) EY262Gsat], and D2R-Cre [MMRRC Tg (Drd2-cre) ER44Gsat] mouse lines were acquired from the Mutant Mouse Regional Resource Center (MMRRC; Davis, California). These mice were backcrossed with C57BL/6J mice. C57BL/6J mice were purchased from Beijing Vital River (Beijing). All animals were housed with their littermates in a dedicated housing room under a 12/12 h light/dark cycle, and food and water were available *ad libitum*. For all experiments, adult male mice (2–3 months old) were used.

### Virus Information

All viral tools used in this study were packaged and provided by BrainVTA (BrainVTA Co., Ltd., Wuhan, China). The detailed production and concentration procedures for modified RV were previously described (Osakada et al., 2011). The final titer of RV-EnvA-ΔG-dsRed was 2 × 10^8^ infecting units per milliliter. For adeno-associated viruses (AAVs), AAV9-EF1a-FLEX-EGFP-2a-TVA and AAV9-EF1a-FLEX-RG were packaged into 2/9 serotypes with final titers at 1–5 × 10^12^ genome copies per milliliter.

### Surgery and Viral Injections

Before virus injection, experimental mice were anesthetized with 2% chloral hydrate, 10% ethylurethane, and 1.7 mg/mL xylazine in a 0.9% NaCl mixture (0.09 ml/10 g body weight, i.p.) and mounted in a stereotaxic holder (item: 68030, RWD, Shenzhen, China). After the skulls of the experimental mice were adjusted to be parallel to the reference panel, a craniotomy (∼0.5 mm diameter) was made above the targeted areas with a dental drill. For cell-type-specific tracing, 25–30 nl of helper AAV (AAV9-EF1a-FLEX-EGFP-2a-TVA and AAV9-EF1a-FLEX-RG mixed at a 1:2 ratio of viral particles) was stereotactically injected into the NAc core (coordinates in mm: AP + 1.34, ML 1.10, DV:-4.3) or the NAc shell (coordinates in mm: AP + 1.34, ML 0.55, DV:-4.7) using a glass micropipette connected to a syringe pump (Item: 53311, Quintessential stereotaxic injector, Stoelting, United States). The micropipette was held in place for 10 min after the injection before being slowly retracted from the brain. Two weeks after helper AAV injection, 150–200 nl of RV-EnvA-ΔG-dsRed was injected into the same location under biosafety level 2 conditions through the same procedure mentioned above.

### Histology and Immunostaining

One week after injection of RV, mice were perfused intracardially with 0.01 M phosphate-buffered saline (PBS), followed by 4% paraformaldehyde (PFA) in 0.01 M PBS. Mouse brains were carefully removed and placed in 4% PFA solution overnight for postfixation. The procedures for agarose embedding were generally performed as reported previously (Sallee and Russell, 1993; Jiang et al., 2017). Briefly, after postfixation, each intact brain was washed with PBS for 4 h and dehydrated in 10% sucrose in PBS overnight. The brains were slightly dried and embedded in melted agarose (Sigma-Aldrich, United States) using a silicone mold in a 55°C water bath for 0.5 h. The orientations of the brains were carefully adjusted in the water bath. The mold was then placed at room temperature until the agarose solidified. Subsequently, the brains were separated from the mold and stored in PBS at 4°C until sectioning. Coronal brain slices (50 μm) were prepared using a vibratome (Leica VT1000, Leica Microsystems). Every second section was selected for whole-brain mapping and visualized with a slide scanning microscope (Nikon).

To characterize the starter cells, sections near the injection site were selected for immunofluorescent staining. These sections were blocked with 3% bovine serum albumin (BSA) in PBS-0.3% Triton X-100 for 1 h at room temperature, incubated with the primary rabbit anti-DARPP-32 monoclonal antibody (1:200, Abcam ab40801) overnight at 4°C, washed with PBS (three times for 10 min each at room temperature), incubated with Alexa-Fluor 647 goat anti-rabbit (1:500, Invitrogen A32733) for 2 h at room temperature, incubated with DAPI (1 ng/ml) for 10 min, and finally washed with PBS (five times for 7 min each at room temperature) prior to mounting the slides. We imaged the sections using an LSM 710 inverted confocal microscope (Zeiss).

### Image Analysis

The locations of labeled neurons were manually registered, and the number of cells in each region from both hemispheres was quantified using Fiji (NIH) according to a standard mouse brain atlas (Paxinos and Franklin, 2001). The input from each region was normalized by dividing the number of labeled neurons found in that region by the total number of labeled neurons (excluding the NAc) in each brain to obtain the percentage of total inputs. For statistical analyses, two-tailed unpaired *t*-tests, the Mann–Whitney U test or one-way ANOVA followed by the Bonferroni correction were performed using SPSS (version 13.0). To quantify the similarity in input patterns, we calculated Pearson’s correlation coefficients. Error bars indicate the SEM.

## Results

### Strategies for Tracing Monosynaptic Inputs to D1R- and D2R-MSNs Within Different NAc Subregions

**FIGURE 1:**
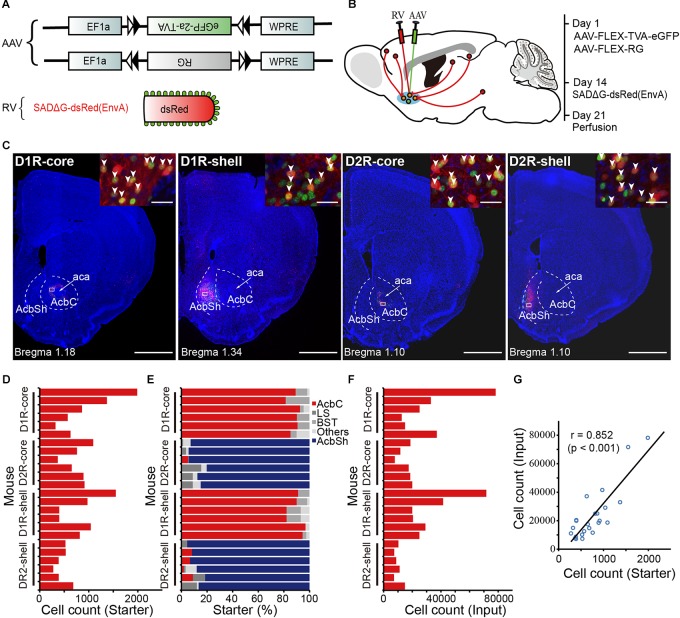
Experimental strategy for identification of monosynaptic inputs to D1R- and D2R-MSNs in different NAc subregions. **(A)** Recombinant AAV strains and RV. **(B)** Experimental design. **(C)** Representative coronal brain sections near the injection sites. Blue indicates cell nuclear staining with DAPI. Scale bar, 1000 μm. Inset, enlarged view of the area in white the box showing starter cells (yellow, expressing both eGFP and dsRed, indicated by white arrowheads). Scale bar, 30 μm. AcbC, nucleus accumbens, core (NAc core); aca, anterior commissure, anterior part; AcbSh, nucleus accumbens, shell (NAc shell). **(D)** Numbers of starter neurons in the individual animals. **(E)** Proportions of labeled starter neurons in injection regions. LS, lateral septal nucleus; BST, bed nucleus of the stria terminalis. **(F)** Numbers of transsynaptically labeled neurons, i.e., input neurons in the individual animals. **(G)** A linear relationship was detected between the number of starter and input neurons.

Our aim is to trace, determine and compare the direct inputs to D1R- and D2R- MSNs in the NAc core and shell (hereafter called D1R-core, D1R-shell, D2R-core, and D2R-shell neurons). We genetically targeted different projection neuron subpopulations in the NAc by using D1R- and D2R-Cre mouse lines, which have been shown to label D1R- and D2R-expressing neuron populations with high specificity (Gong et al., 2007). To demonstrate the whole-brain monosynaptic inputs for each cell type, we used a retrograde transsynaptic tracing system based on a modified RV, SAD-ΔG-dsRed (EnvA) (Wickersham et al., 2007). First, we coexpressed the avian receptor TVA and rabies glycoprotein G (RG) specifically in each cell type, which was achieved through injection of two Cre-inducible AAV vectors (AAV9-EF1a-DIO-eGFP-TVA and AAV9-EF1a-DIO-RG) into D1R- or D2R-Cre mice. Injections were targeted to either the NAc core or the NAc shell. Two weeks later, we injected SAD-ΔG-dsRed(EnvA) into the same area (**Figures 1A,B**). This modified RV can infect only those cells expressing TVA and requires RG to spread retrogradely to presynaptic cells. Mice were perfused 1 week later; the whole brain was sectioned at 50 μm, and every second section was selected for further analysis.

The starter cells were identified based on the coexpression of TVA-eGFP and dsRed. We observed a large number of double-labeled neurons near the injection sites (**Figures 1C,D**). Combined with DARPP-32 immunostaining, a reliable marker of both populations of MSNs (Ouimet et al., 1984; Matamales et al., 2009), we found that almost all eGFP- and dsRed-double-positive neurons (starter cells) were also DARPP-32-positive, confirming that they are MSNs, in both D1R-Cre and D2R-Cre mice (**Supplementary Figure S1**). For each group, the location of the vast majority of starter cells was restricted to the injected subregion of the NAc (**Figure 1C**), although we found a small number of eGFP- and dsRed-double-positive neurons in neighboring nuclei: the lateral septal nucleus (LS), bed nucleus of the stria terminalis (BST), anterior olfactory nucleus (AON), and caudate putamen (CPu). However, these neurons made up a small fraction of total starter neurons in most animals (**Figure 1E**). We found 7336–78192 dsRed-labeled presynaptic neurons in each brain (*n* = 24) (**Figure 1F**). Variation of the cell numbers across animals was partly due to different injection volumes. Nevertheless, the numbers of transsynaptically labeled neurons had a linear relationship with the numbers of starter neurons (**Figure 1G**).

**FIGURE 2:**
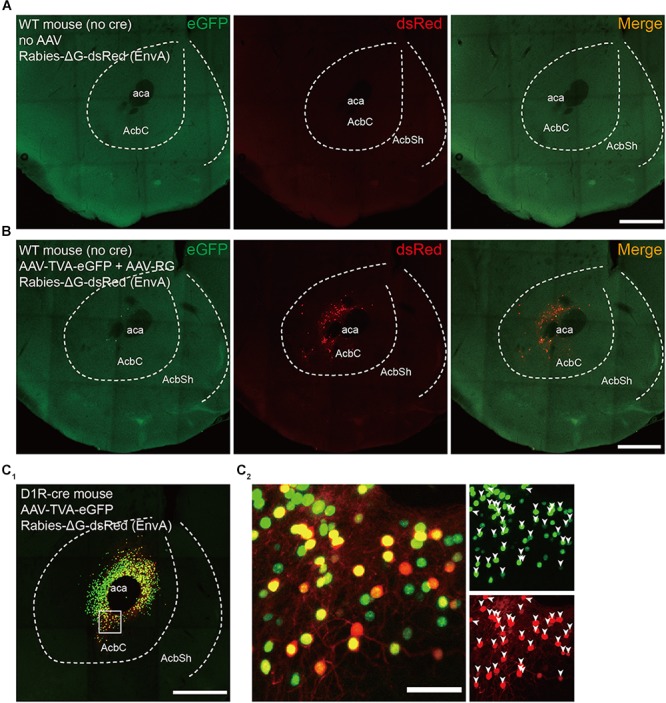
Control experiments for rabies-mediated transsynaptic tracing. **(A)** Injection of RV without prior AAV injection resulted in no dsRed-labeled neurons, indicating the dependence of the RV infection on AAV-induced expression of TVA. Scale bar, 500 μm. **(B)** Injecting AAV-DIO-EGFP-TVA, AAV-DIO-RG, and RV into the wild-type mice led to a low level of nonspecific labeled RV neurons that expressed dsRed at the injection site. Scale bar, 500 μm. **(C_1_)** Injection of AAV-DIO-EGFP-TVA and RV without prior AAV-DIO-RG injection in the D1R-Cre mice resulted in no DsRed-labeled input neurons. Scale bar, 500 μm. **(C_2_)** Enlarged view of the boxed region in panel **(C_1_)**; all dsRed-positive neurons coexpressed eGFP. Scale bar, 50 μm.

A number of control experiments were conducted to define the specificity of our tracing approach. We first injected SAD-ΔG-dsRed (EnvA) into the NAc core of wild-type animals (i.e., Cre-negative) without prior AAV injection. This treatment resulted in no dsRed-labeled neurons (**Figure 2A**), indicating the dependence of the RV infection on AAV-induced expression of TVA. Next, we repeated the retrograde transsynaptic tracing strategy in wild-type animals. Consistent with previous studies using similar methods (Watabe-Uchida et al., 2012; Weissbourd et al., 2014; Do et al., 2016), slight local background infection by RV was observed (**Figure 2B**), most likely due to the leaky expression of a low level of TVA. Note that all these non-specifically labeled neurons were found near the injection sites, within the NAc. Thus, to rule out the impact of this nonspecifically labeling on the mapping of long-range inputs, the following analysis excluded data originating from the NAc. Furthermore, to verify that the transsynaptic spread is under the tight control of RG expression, we injected only AAV9-EF1a-DIO-eGFP-TVA and SAD-ΔG-dsRed (EnvA) into the NAc core of D1R-Cre mice. A large number of labeled neurons were found near the injection site, and almost all dsRed-positive neurons coexpressed eGFP (**Figures 2C_1_,C_2_**). In contrast, we did not detect any labeled neurons outside the NAc (**Figure 2C_1_**). Together, these results demonstrated that our tracing approach was suitable for mapping the long-range monosynaptic inputs to the NAc and that labeled neurons outside the injection sites represent monosynaptic inputs to projection neurons in the NAc.

### Long-Range Cell-Type-Specific Inputs to the NAc Core and Shell

**FIGURE 3:**
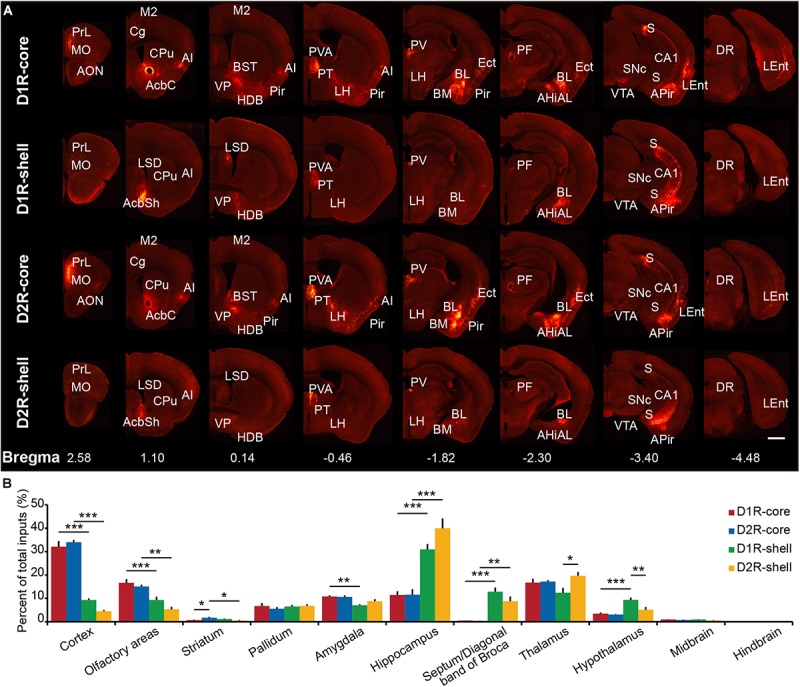
Overview of whole brain input to D1R-MSNs and D2R-MSNs in different NAc subregions. **(A)** Representative coronal sections showing labeling of monosynaptic inputs to D1R-core, D1R-shell, D2R-core, and D2R-shell neurons. Only the side ipsilateral to the injection site is shown. Scale bar, 1 mm. **(B)** Proportions of total inputs from 11 brain areas. Mean ± SEM (*n* = 6 mice each for the D1R-core, D1R-shell, D2R-core, and D2R- shell groups). ^∗∗∗^*p* < 0.001, ^∗∗^*p* < 0.01, and ^∗^*p* < 0.05. Only significant differences between the same cell type in different subregions or between different cell types in the same subregions are marked; one-way ANOVA with Bonferroni correction.

Representative coronal images from the transsynaptic tracing of inputs to each group show the whole-brain distribution of dsRed-labeled neurons (**Figure 3A**). We found that NAc (including core and shell) neurons integrate inputs from diverse brain regions, from the anterior neocortex to specific midbrain areas (**Figure 3A**). The dsRed-labeled neurons were predominantly found ipsilateral to the injection site, although sparser labeling was also observed in the contralateral hemisphere (data not shown). To quantify the distributions of long-range monosynaptic inputs, we counted the number of dsRed-labeled neurons within individual regions of each brain. Since the total number of labeled neurons varied across brain samples, to directly compare between experiments, we normalized the data in each region by the total number of labeled neurons in each brain (**Supplementary Table S1**).

When the brain was divided into 11 major brain areas (**Figure 3B**), the hippocampus contributed most of the long-range inputs to the NAc, followed by the cortex, thalamus, olfactory areas, amygdala, pallidum, hypothalamus, septum/diagonal band of Broca, striatum (excluding the NAc), midbrain, and hindbrain (**Figure 2B**). Even at this gross anatomical level, the pattern of inputs targeting the NAc core versus NAc shell shows a notable difference, particularly in the cortex and hippocampus (**Figure 2B**): the cortex preferentially projects to the NAc core, whereas the hippocampus preferentially projects to the NAc shell.

**FIGURE 4:**
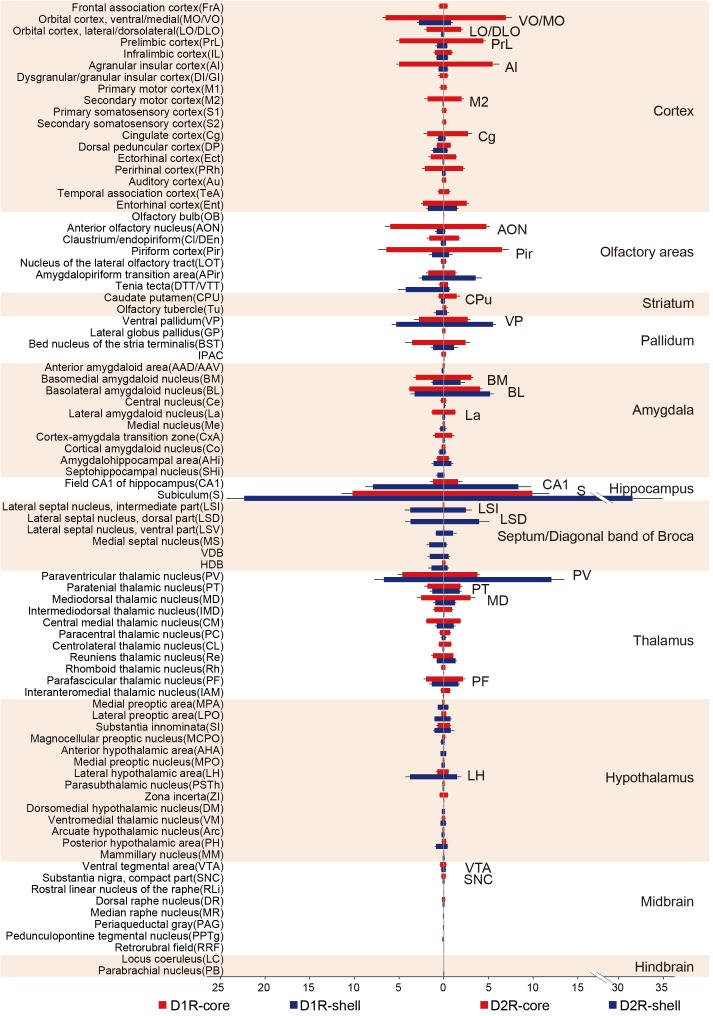
Quantitative analysis of the proportions of whole-brain input to D1R-MSNs and D2R-MSNs in different NAc subregions. (Left) Monosynaptic inputs to D1R-MSNs in the NAc core (red) and NAc shell (blue). (Right) Monosynaptic inputs to D2R-MSNs in the NAc core (red) and NAc shell (blue). Mean ± SEM (*n* = 6 mice for each group).

To investigate input tracing in more detail, we further divided the brain into 84 regions. **Figure 4** displays the proportion of input neurons in specific regions. Despite the functional differences of D1R-MSNs and D2R-MSNs in the NAc, the overall input distribution patterns of D1R-MSNs (**Figure 4**, left) and D2R-MSNs (**Figure 4**, right) were similar regardless of whether they were located in the NAc core or NAc shell. All regions projecting to D1R-MSNs also provided direct inputs to D2R-MSNs and vice versa. For both D1R-MSNs and D2R-MSNs, most of the inputs to the NAc shell were concentrated in a few brain regions (**Figure 4**, blue), and conversely, inputs to the NAc core were broadly scattered (**Figure 4**, red). For example, the subiculum (S), paraventricular thalamic nucleus (PV), hippocampal CA1 region and ventral pallidum (VP) were the four major presynaptic regions that projected to the NAc shell, and inputs from these four regions accounted for ∼50% of total inputs to the NAc shell (42.3 ± 7.6% for D1R-MSNs; 57.5 ± 13.0% for D2R-MSNs). These results suggested that both types of MSNs in the NAc might be regulated by many common upstream regions, whereas the NAc core might integrate information from a broader brain area than the NAc shell.

### Multiple Nuclei Provide Significant Contrasting Inputs to the NAc Core and Shell

As mentioned above, the overall input distribution patterns of D1R-MSNs and D2R-MSNs in both NAc subregions show a high degree of similarity; therefore, we will consider these two types of neurons in each subregion together in the next analysis.

**FIGURE 5:**
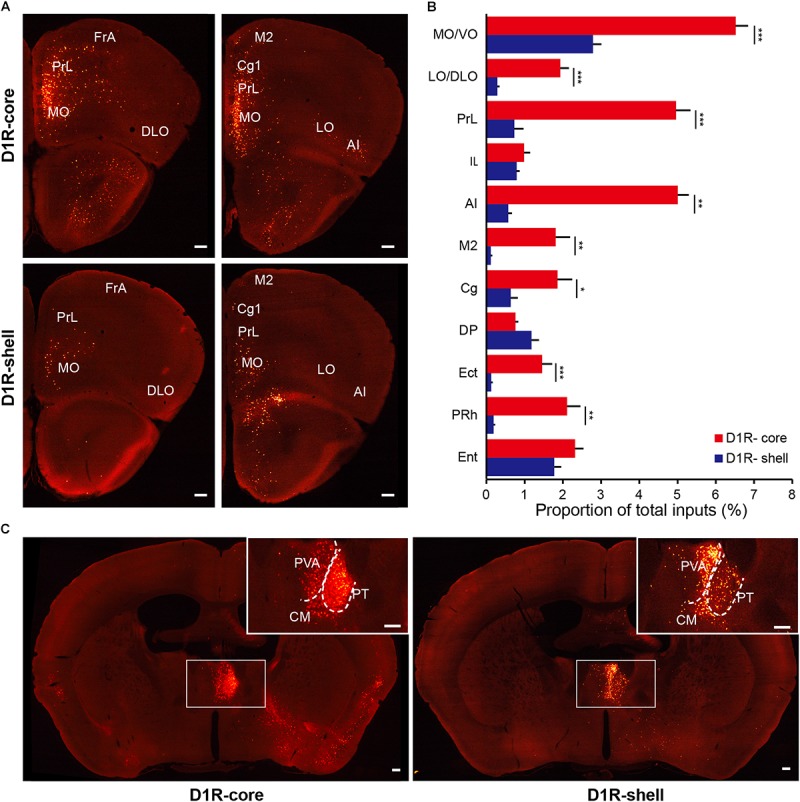
Monosynaptic inputs to D1R-MSNs from the cortex and thalamus. **(A)** Distributions of anterior cortex inputs to D1R-MSNs in the NAc core (upper panels) and shell (lower panels). Scale bar, 200 μm. **(B)** Proportion of total inputs to D1R-MSNs in the NAc core (red) and shell (blue) from 11 cortical areas that contained relatively large numbers of input neurons (>1% in at least one experimental group). Mean ± SEM (*n* = 6 mice for each group). ^∗∗∗^*p* < 0.001, ^∗∗^*p* < 0.01, and ^∗^*p* < 0.05, two-tailed unpaired *t*-test or Mann–Whitney U test. **(C)** Distributions of thalamic inputs to D1R-MSNs in the NAc core (right) and shell (left). Inset, enlarged view of the area in the white box showing spatially separated labeling. Scale bar, 200 μm.

**FIGURE 6:**
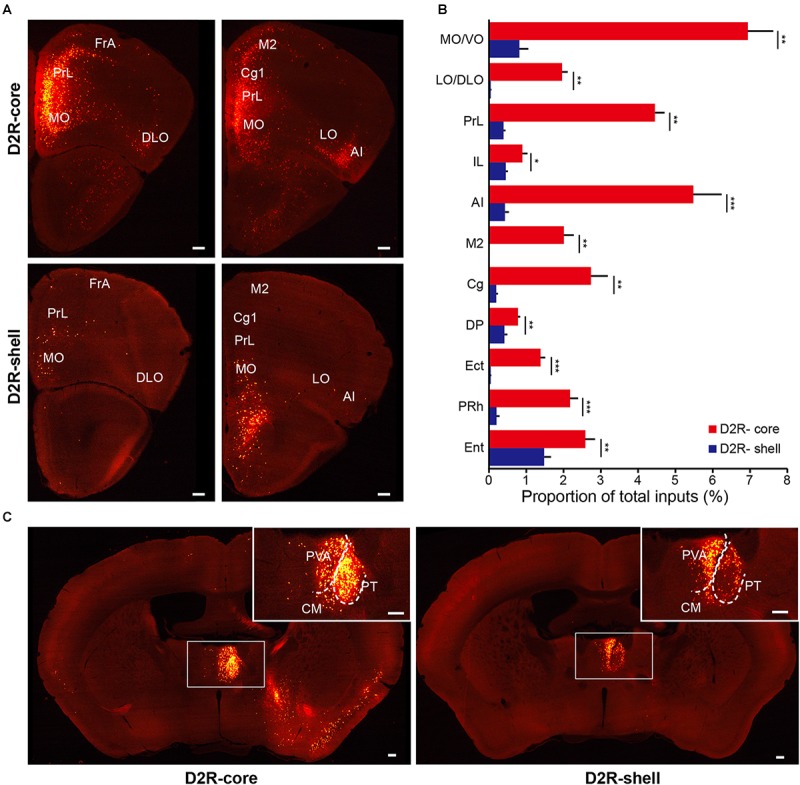
Monosynaptic inputs to D2R-MSNs from the cortex and thalamus. **(A)** Distributions of anterior cortex inputs to D2R-MSNs in the NAc core (upper panels) and shell (lower panels). Scale bar, 200 μm. **(B)** Proportion of total inputs to D2R-MSNs in the NAc core (red) and shell (blue) from 11 cortical areas that contained relatively large numbers of input neurons (>1% in at least one experimental group). Mean ± SEM (*n* = 6 mice for each group). ^∗∗∗^*p* < 0.001, ^∗∗^*p* < 0.01, and ^∗^*p* < 0.05, two-tailed unpaired *t*-test or Mann–Whitney U test. **(C)** Distributions of thalamic inputs to D2R-MSNs in the NAc core (right) and shell (left). Inset, enlarged view of the area in the white box showing spatially separated labeling. Scale bar, 200 μm.

When the virus injection was targeted to the NAc core, the labeled neurons were widely distributed across cortical areas. In contrast, only sparse labeling was found in cortical areas when we targeted the injection to the NAc shell (**Figures 5A,B**, **6A,B**; also see **Figure 4**). This difference was especially pronounced in the anterior cortex. For example, we found dense labeling in the medial and ventral orbital cortex (MO/VO), agranular insular cortex (AI) and prelimbic cortex (PrL) when tracing inputs to the NAc core, with significantly fewer labeled neurons when tracing inputs to the NAc shell. In addition, other anterior cortical areas, including the lateral and dorsolateral orbital cortex (LO/DLO), cingulate cortex (Cg) and secondary motor cortex (M2), also preferentially project to the NAc core (**Figures 5A,B**, **6A,B**).

Among the rostral forebrain areas, NAc core injection led to dense labeling in the AON and piriform cortex (Pir), whereas NAc shell injection caused the densest labeling in the tenia tecta (DTT/VTT) (**Figure 4**). Despite being near the NAc (the injection site), the dorsal striatum, which prominently includes the CPu, receives only a small fraction of the total inputs in both NAc core- and shell-targeted cases. In fact, labeled neurons in this structure may be “spill-over” from the NAc (**Figures 3A**, **4**). That is, labeled neurons were often present at the border between the CPu and NAc, whereas the center of the CPu showed very sparse or no labeling. Conventional tracing studies have identified a strong reciprocal connection between the NAc and VP (Groenewegen et al., 1999). In accordance with these studies, we found that the VP provides many inputs to both the NAc core and NAc shell, with a slight preference for the NAc shell (**Figure 4**).

In the amygdala, two subdivisions account for nearly all amygdala projections to the NAc: the basolateral amygdaloid nucleus (BL, including the anterior, posterior and ventral parts of the basolateral nucleus, BLA/BLP/BLV) and basomedial amygdaloid nucleus (BM, including the anterior and posterior parts of the basomedial nucleus, BMA/BMP) (**Figures 3A**, **4**). While the BL makes up a similar proportion of the total inputs to the NAc core and shell, the BM makes up a significantly larger proportion of inputs to the NAc core (**Figure 4**). We found a sparser distribution of labeled neurons in the central nucleus (Ce) and medial nucleus (Me) for both NAc core- and shell-targeted cases (**Figure 4**). Interestingly, the lateral amygdaloid nucleus (La) shows a notable preference to project to the NAc core (**Figure 4**).

Consistent with conventional retrograde tracing studies (Brog et al., 1993), we found that the LS, particularly the ventral and dorsal divisions (LSV/LSD), sends prominent monosynaptic inputs to the NAc shell but has very sparse connectivity with the NAc core (**Figures 3A**, **4**). The connectivity between the hippocampus and the NAc has attracted great interest as a circuit involved in spatial navigation and recognition of novelty (Floresco et al., 1997; Ito et al., 2008). Here, we found many inputs from the hippocampus. Indeed, the S contained the largest numbers of total labeled neurons for both NAc core- and shell-targeted cases, although with significantly more projections to the NAc shell (**Figures 3A**, **4**). The NAc shell also received dense inputs from the CA1 region of the hippocampus, whereas the NAc core received relatively few inputs from this region (**Figures 3A**, **4**).

In the thalamus, the projections to the NAc core and shell were roughly spatially segregated. For example, more dorsal and medial structures, such as the PV, particularly the anterior dorsal part, project preferentially to the NAc shell. In contrast, more ventral and lateral structures, such as the paratenial thalamic nucleus (PT) and mediodorsal thalamic nucleus (MD), show a notable trend toward projecting to the NAc core (**Figures 4**, **5C**, **6C**). These results correspond well to previous experiments using traditional retrograde tracers to label inputs to different subregions of NAc (Brog et al., 1993). Other thalamic nuclei, including the intermediodorsal (IMD), central medial (CM), paracentral (PC), and centrolateral thalamic nuclei (CL) also send more inputs to the NAc core than to the NAc shell (**Figure 4**). Additionally, we detected a large number of labeled neurons in the parafascicular nucleus (PF) for both NAc core- and shell-targeted cases (**Figures 3A**, **4**).

Hypothalamic nuclei play a critical role in the regulation of feeding and circadian rhythms (Saper, 2006). Many hypothalamic areas provide scattered inputs to both the NAc core and NAc shell with similar patterns (**Figure 4**). We found that there was a slight preference for hypothalamic nuclei, such as the lateral preoptic area (LPO), medial preoptic area (MPA), and substantia innominata (SI), to project to the NAc core. In these areas, the lateral hypothalamic area (LH) is unique in that its inputs to the NAc are strongly biased toward the shell. Interestingly, our cell-type-specific tracing revealed that the LH preferentially targets D1R-MSNs over D2R-MSNs in the NAc shell (see section “Discussion”).

There were very few inputs from the midbrain and hindbrain to either the NAc core or the NAc shell (**Figure 4**). The ventral tegmental area (VTA) and substantia nigra pars compacta (SNc), known to contain a high density of dopaminergic neurons, provided the major midbrain inputs to both NAc subregions. Some labeling was also sparsely distributed in the dorsal raphe nucleus (DR), median raphe nucleus (MnR), rostral linear nucleus of the raphe (RLi), periaqueductal gray (PAG), pedunculopontine tegmental nucleus (PPTg), retrorubral field (RRF), locus coeruleus (LC), and parabrachial nucleus (PB), suggesting rather weak inputs from these structures to the NAc.

In summary, these results show that although inputs to the NAc core and shell originate from largely overlapping areas, many cortical and subcortical nuclei provide significant contrasting inputs to these two subregions.

### Comparison Between Inputs to D1R- and D2R-MSNs in Different NAc Subregions

**FIGURE 7:**
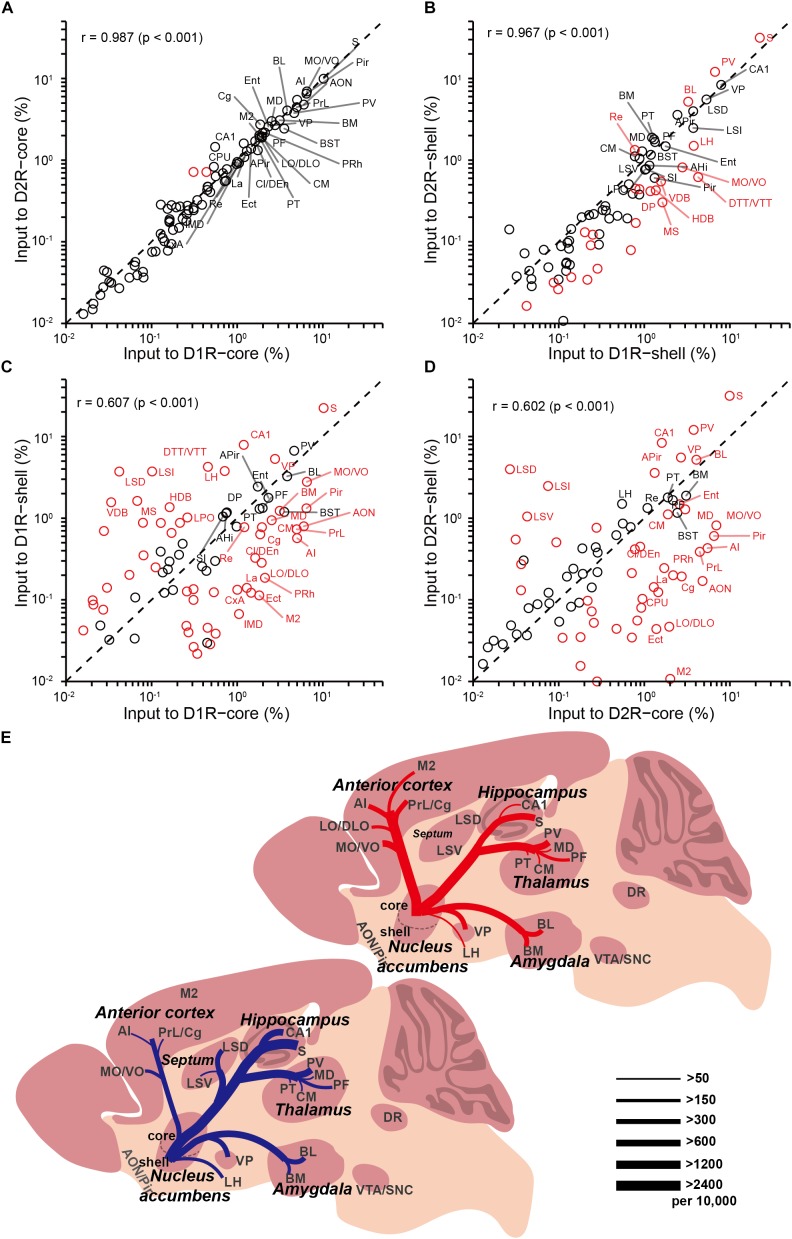
Comparisons of input distributions across the four groups. **(A)** Comparison between inputs to D1R-core and D2R-core neurons. **(B)** Comparison between inputs to D1R-shell and D2R-shell neurons. **(C)** Comparison between inputs to D1R-core and D1R-shell neurons. **(D)** Comparison between inputs to D2R-core and D2R-shell neurons. Values are the means of the percent of total inputs from each region. Red circles indicate significant differences (*p* < 0.05, two-tailed unpaired *t*-test or Mann–Whitney U test). *r*, Pearson’s correlation coefficient. **(E)** Summary of selected prominent monosynaptic inputs to the NAc core (top, red) and shell (bottom, blue), considering D1R-MSNs and D2R-MSNs together. Line thickness represents the number of inputs.

In our analysis of long-range tracing, we found that although the input patterns to D1R-MSNs and D2R-MSNs are highly similar, the input patterns to different subregions of the NAc differ significantly. To validate our previous findings, we quantified the similarities and differences in the distribution patterns of inputs to the four groups (D1R-core, D1R-shell, D2R-core, and D2R-shell neurons). **Figures 7A–D** show the correlation coefficients for all pairs (Ogawa et al., 2014). Each open circle in the scatter plots represents one brain region (significant differences in red, *p* < 0.05), and the diagonal represents the same input proportion for each pair. Comparing the input patterns of D1R- and D2R-MSNs, we found that the vast majority of the brain regions, represented by the open circles centered around the diagonals, showed that they provided similar numbers of inputs to D1R-MSNs and D2R-MSNs in both NAc subregions (**Figures 7A,B**). In the NAc core- and shell-targeted cases, we found that only 2 and 24 of 84 brain regions, respectively, provided significantly different inputs to D1R-MSNs and D2R-MSNs. The correlation coefficients between inputs to D1R-MSNs and D2R-MSNs in both the NAc core and the NAc shell were extremely high (correlation coefficient, *r* = 0.987 for D1R-core versus D2R-core; *r* = 0.967 for D1R-shell versus D2R-shell, *p* < 0.001; **Figures 7A,B**), confirming their high degree of similarity as observed earlier. On the other hand, when we computed the correlation coefficients between inputs to the same cell type in different NAc subregions, we found relatively low correlation coefficients (*r* = 0.607 for D1R-core versus D1R-shell; *r* = 0.602 for D2R-core versus D2R-shell, *p* < 0.001; **Figures 7C,D**). The open circles representing brain regions were relatively scattered from the diagonal. The D1R-MSNs in the NAc core and shell received significantly different inputs from 57 of 84 brain regions (**Figure 7C**). There were 52 of 84 brain regions that provided significantly different inputs to D2-MSNs in different NAc subregions (**Figure 7D**). The results are presented in **Table 1**.

**Table 1:** Brain areas providing significantly different inputs to each comparison pair.

Brain area	For D1R-MSNs		For D2R-MSNs		Within NAc core		Within NAc shell	
	Core^pre^	Shell^pre^	Core^pre^	Shell^pre^	D1R^pre^	D2R^pre^	D1R^pre^	D2R^pre^
MO/VO	^∗∗∗^		^∗∗^				^∗∗∗^	
LO/DLO	^∗∗∗^		^∗∗^				^∗∗^	
PrL	^∗∗∗^		^∗∗^					
AI	^∗∗^		^∗∗∗^					
M2	^∗∗^		^∗∗^					
Cg	^∗^		^∗∗^					
DP			^∗∗^				^∗∗^	
Ect	^∗∗∗^		^∗∗∗^					
PRh	^∗∗^		^∗∗^					
Ent			^∗∗^					
AON	^∗∗^		^∗∗^				^∗∗^	
Cl/DEn	^∗∗^		^∗∗^					
Pir	^∗∗^		^∗∗∗^					
APir				^∗∗^				
DTT/VTT		^∗∗^					^∗∗^	
CPU			^∗∗^					
VP		^∗∗^		^∗∗∗^				
BST								
BM	^∗∗∗^							
BL				^∗^				^∗^
La	^∗∗^		^∗∗∗^					
CxA	^∗∗^		^∗∗^					
AHi								
CA1		^∗∗∗^		^∗∗^				
S		^∗∗∗^		^∗∗∗^				^∗^
LSI		^∗∗^		^∗∗^				
LSD		^∗∗^		^∗∗^				
LSV		^∗∗∗^		^∗∗^				
MS		^∗∗^					^∗∗^	
VDB		^∗∗^		^∗^			^∗^	
HDB		^∗∗^					^∗^		
PV				^∗∗^				^∗^
PT								
MD	^∗^		^∗^					
IMD	^∗∗^		^∗∗^					
CM	^∗∗∗^		^∗∗^					
Re	^∗^							^∗∗^
PF								
LPO		^∗∗∗^		^∗∗^				
SI								
LH		^∗∗∗^					^∗∗^	

*Only those areas that make up more than 1% of total inputs in at least one of the four starter groups are listed. The symbol “^∗^” indicates significantly different inputs to each comparison pair, ^∗∗∗^*p* < 0.001, ^∗∗^*p* < 0.01, and ^∗^*p* < 0.05, two-tailed unpaired *t*-test or Mann–Whitney U test*.

## Discussion

Using rabies-mediated transsynaptic tracing, we mapped the whole-brain monosynaptic inputs to the two main projection cell types (D1R-MSNs and D2R-MSNs) in different NAc subregions. Our experiments confirmed many previously demonstrated connections, but with cell-type specificity and quantitative analyses. Our data showed an extremely high degree of overall similarity between inputs to D1R-MSNs and D2R-MSNs in both NAc subregions. However, the input patterns to different NAc subregions differ significantly (**Figure 7E**). Both D1R- and D2R-MSNs in the NAc core are more likely than those in the shell to receive monosynaptic inputs from the anterior cortex, while D1R-MSNs in the NAc shell receive disproportionally higher input from the LH. Through quantitative comparisons, we identified a list of brain nuclei that provided significantly different inputs to each comparison pair (**Table 1**).

Accumulated evidence has indicated that D1R-MSNs and D2R-MSNs in the NAc (core + shell) play differential and often opposing roles in reinforcement learning (Lobo et al., 2010; Yawata et al., 2012). A recent study combining an AAV-based anterograde tracing approach and optogenetics demonstrated the classical separation of D1R-MSNs in the direct pathway and D2R-MSNs in the indirect pathway did not necessarily apply in NAc (Kupchik et al., 2015). However, the organizing principles of afferents to these two MSN subtypes in the NAc remain unclear. Here, we found that D1R-MSNs and D2R-MSNs in both NAc subregions received quantitatively similar patterns of inputs, suggesting that these two MSN subtypes integrate information from largely overlapping brain areas. Interestingly, our result is similar to the results of recent transsynaptic tracing studies in the DR (Weissbourd et al., 2014), the VTA (Beier et al., 2015), the basal forebrain (BF) (Do et al., 2016), the arcuate nucleus (ARC) (Wang et al., 2015), the dorsal striatum (Guo et al., 2015), and the medial part of the olfactory tubercle (OT) (Zhang et al., 2017), where different cell types within the same brain structures receive similar overall inputs from diverse brain regions. Given the extensive interconnection between local axonal collaterals of D1R- and D2R-MSNs (Kawaguchi et al., 1989; Taverna et al., 2008; Chuhma et al., 2011), the coinnervation of these two MSN subtypes may reflect complex computations by striatal microcircuits and a need to modulate MSN activity through collateral inhibition (Taverna et al., 2008). It has been reported that D1 and D2 receptors exhibit a marked difference in their dopamine binding affinity (Maeno, 1982; Richfield et al., 1989); thus, phasic and tonic firing of dopamine neurons differentially modulate D1 and D2 receptors, respectively (Grace et al., 2007; Hikosaka, 2007). These characteristic features of dopamine transmission may result in the distinct functions mediated by D1R- and D2R-MSNs despite their shared upstream dopamine pathway (Hikida et al., 2010). However, given the vast differences in the expression profiles of functional proteins and intracellular signaling molecules between D1R- and D2R-MSNs (Gerfen et al., 1990; Grace et al., 2007; Hikosaka, 2007; Heiman et al., 2008), it remains to be determined whether these two MSN subtypes in the NAc are connected by separate subpopulations within the overlapping upstream brain areas. Future research will require the development of new technologies that allow us to label the presynaptic partners of separate cell types with different colors within the same animal.

The transsynaptic tracing strategy allowed us to quantitatively compare inputs to different cell types of different NAc subregions. Although inputs to D1R-MSNs and D2R-MSNs in the NAc show a high degree of similarity, we identified multiple nuclei that provide significant biased inputs to different NAc subregions. The difference observed in the anatomical connections of the NAc core and shell likely underlies their distinct behavioral functions. For example, we found that many anterior cortical areas (including the VO/MO, LO/DLO, AI, PrL, M2, and Cg) were preferentially labeled when starter cells were located in the NAc core but not the shell. Interestingly, most of these structures have long been implicated in value-based decision making (Hauber and Sommer, 2009; Sul et al., 2010, 2011). Thus, our results may provide explanations for the prominent role of the NAc core rather than the shell in instigating approach behavior toward motivationally relevant stimuli.

The NAc shell-to-LH pathway is well documented to play an important role in feeding control (Kelley et al., 2005; Urstadt et al., 2013; O’Connor et al., 2015). For example, pharmacological inhibition of the NAc shell triggers increased feeding that can be prevented by concomitant infusion of a gamma-aminobutyric acid type A (GABAA) receptor agonist into the LH (Urstadt et al., 2013). A recent study showed that D1R-MSNs in the NAc shell provide the predominant source of accumbal input to the LH (O’Connor et al., 2015). In our tracing study, we found that the LH also projects preferentially to the NAc shell, consistent with previous findings (Brog et al., 1993). On the other hand, although the input from the LH is strongly biased toward the NAc shell as opposed to the core, we extend the previous findings by showing that the LH projects preferentially to D1R-MSNs, over D2R-MSNs in the NAc shell. Although we have not yet determined the functional significance of this reverse pathway, our findings provide an anatomical basis that may help elucidate the computational mechanisms underlying the regulation of energy homeostasis.

It is worth mentioning that we detected only a small proportion of total inputs from the VTA and SNc, which contain the major dopaminergic populations, as well as from the DR and MnR, the sites of the major serotonergic populations. These results appear to differ from some traditional tracing studies indicating that the striatum (the CPu and NAc) receives substantial dopaminergic (Fallon and Moore, 1978; Phillipson and Griffiths, 1985) and serotonergic innervation (Vertes, 1991; Waselus et al., 2006). In contrast, our data are consistent with certain previous studies of the dorsal striatum (Wall et al., 2013; Guo et al., 2015), where rabies-mediated transsynaptic tracing led to weak labeling in the VTA and SNc (Wall et al., 2013), and in the DR and MnR (Guo et al., 2015). It was speculated that this weak transsynaptic RV labeling results from the nature of peculiar synapses between dopaminergic neurons and MSNs (Wall et al., 2013). Similar to dopaminergic projections, many serotonergic projections often form varicosities that lack the junctional complexes classically considered to be the morphological substrate for chemical transmission in the central nervous system (Beaudet and Descarries, 1981; Descarries et al., 2010). Our results, together with previous findings, raise the possibility that these specialized synaptic structures may prevent the RV from spreading from the striatal neurons to the presynaptic partners.

Rabies-mediated transsynaptic tracing has been widely used for elucidating neuronal connectivity. As previously published studies have done before us, we must emphasize again the limitations of this technique (Callaway and Luo, 2015), including the fact that it can label only a fraction of inputs to starter cells and that the number of labeled input neurons does not necessarily reflect functional connectivity strength. For example, one input neuron may form synapses with several MSNs, and one starter cell may also be connected to multiple input neurons. It has been established that the spread of RV may be exclusively transsynaptic (restricted to synapses) (Ugolini, 1995; Callaway, 2008; Ugolini, 2008). However, it is not entirely clear how RV crosses synapses and whether transsynaptic spread of RV is biased toward certain connections. Additionally, although we revealed the input neural circuitries of different NAc subregions, it should be noted that the quantification of inputs depends largely on the location of the virus injection and the spread of the virus. It is difficult to cover the entire NAc subregion while leaving adjacent areas uninfected by the viral injection. To lower the possibility of nonspecific infection, we injected a small volume of virus and used a very slow injection rate to limit the spread of the virus to a small range (see **Figure 1C**). It is possible that other regions that were not targeted in our experiments could show different input patterns.

Another potential caveat arises when we consider that some MSNs coexpress D1 and D2 receptors (D1/D2-MSNs) (Bertran-Gonzalez et al., 2008), posing the possibility that the results of our tracing study using D1R- and D2R-Cre mouse lines contain an overlapping population of starter cells. These D1/D2-MSNs were estimated to account for only 5% of MSNs in the NAc core but up to 17% of MSNs in the NAc shell of mice (Bertran-Gonzalez et al., 2008; Perreault et al., 2011; Gangarossa et al., 2013; Gagnon et al., 2017). Recent studies have shown that this phenomenon of the coexpression of D1 and D2 receptors also exists in rats and nonhuman primates (Perreault et al., 2016; Rico et al., 2017). Furthermore, cholinergic interneurons (CINs), accounting for <5% of the total neuronal population, coexpress D2 receptors in both the striatum and NAc (Le Moine et al., 1990; Pisani et al., 2000; Gagnon et al., 2017), posing the possibility that CINs actually account for a small fraction of the labeled D2-MSNs in our tracing study. It would be intriguing to determine whether the patterns of input organization of these D1/D2-MSNs and CINs are similar to those of D1R-MSNs and D2R-MSNs in the NAc. According to these caveats and limitations, we suggest that our study of inputs to D1R-MSNs and D2R-MSNs in the NAc has revealed trends that likely underestimate the true specificity within NAc circuits. Future studies combining new genetic and viral approaches are necessary to target diverse cell subtypes in the NAc with higher specificity.

In summary, we mapped the organization of inputs to D1R-MSNs and D2R-MSNs in different NAc subregions (core and shell) using transsynaptic retrograde tracing with a modified RV. Although the input distributions of different cell types were highly similar, different NAc subregions had significantly different biased inputs from numerous brain areas. The similarities and differences observed in our study may provide new insight into the diverse functions of the NAc.

## Data Availability

All data generated or analyzed in this study are included in the manuscript.

## Author Contributions

ZL and TX conceived and designed the project. ZL performed the tracing experiments. ZL, GF, AL, and JY performed the whole-brain data acquisition and analyzed the data. ZL and ZC generated the figures. ZL and TX wrote the paper.

## Conflict of Interest Statement

The authors declare that the research was conducted in the absence of any commercial or financial relationships that could be construed as a potential conflict of interest.

## Supplementary Material

FIGURE S1 Confocal images showing almost all eGFP- and dsRed-double-positive neurons (starter cells) were also DARPP-32-positive in D1R-Cre mice (top, arrowheads) and D2R-Cre mice (bottom, arrowheads). Scale bar, 10 μm.

TABLE S1 Raw data used for quantification.
