# Supplementary figures and images for: Cell-Type-Specific Afferent Innervation of the Nucleus Accumbens Core and Shell
Source: Front Neuroanat. 2018 Oct 16;12:84. doi: 10.3389/fnana.2018.00084 (PMC6232828; doi:10.3389/fnana.2018.00084)

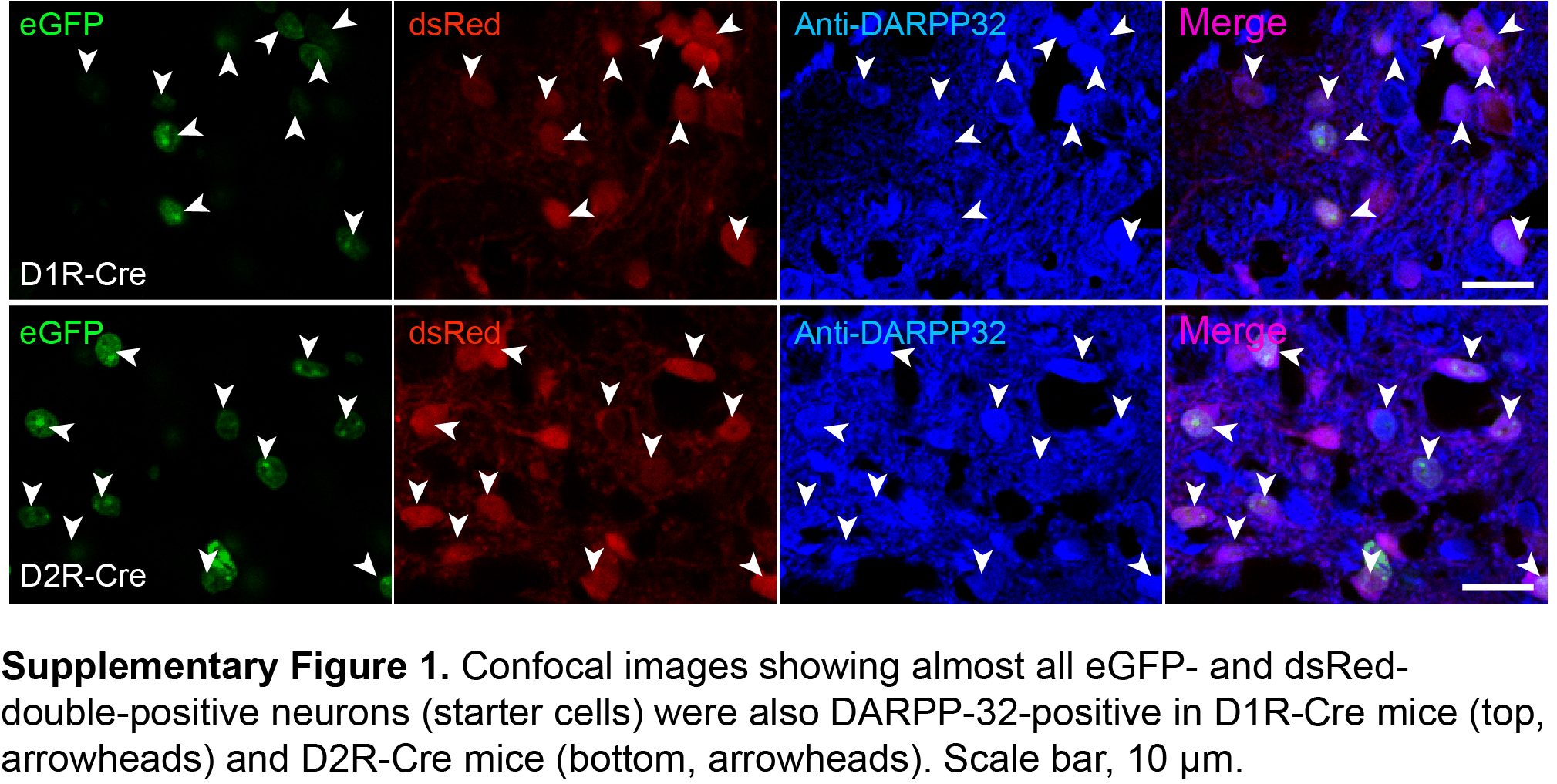

Supplement: FIGURE S1 — Confocal images showing almost all eGFP- and dsRed-double-positive neurons (starter cells) were also DARPP-32-positive in D1R-Cre mice (top, arrowheads) and D2R-Cre mice (bottom, arrowheads). Scale bar, 10 μm. [file Image_1.TIF]
